# Oxybenzone (Benzophenone-3) Induces Antioxidant Responses and DNA Strand Breakage in *Aiptasia pallida*, a Coral Research Model Organism

**DOI:** 10.3390/toxics14070594

**Published:** 2026-07-06

**Authors:** Ezekiel Tosin Babatunde, Manoj Chand, Nevannah Harlan, Claire Korte, Kevin R. Tucker, Christopher Theodorakis

**Affiliations:** 1 Department of Environmental Sciences, Southern Illinois University Edwardsville, 44 Circle Drive, Edwardsville, IL 62026, USA; ebabatu@siue.edu (E.T.B.); manocha@siue.edu (M.C.); 2 Shimadzu Innovation Laboratory, Department of Chemistry, Southern Illinois University Edwardsville, 44 Circle Drive, Edwardsville, IL 62026, USA; neharla@siue.edu (N.H.); clkorte@siue.edu (C.K.); kevtuck@siue.edu (K.R.T.); 3 Department of Biological Sciences, Southern Illinois University Edwardsville, 44 Circle Drive, Edwardsville, IL 62026, USA

**Keywords:** oxybenzone, *Aiptasia pallida*, catalase, superoxide dismutase, glutathione, comet assay, genotoxicity, oxidative stress, benzophenone-3, coral reef, UV filter

## Abstract

The toxicity of oxybenzone (benzophenone-3; BP-3), a UV filter present in coral reef waters at toxic concentrations, has been incompletely characterized for oxidative stress responses in adult cnidarians. In order to examine this, *Aiptasia pallida* (an anemone commonly used as a coral research model) was exposed to oxybenzone at concentrations of 0.02, 0.2, 2.0, and 3.0 mg/L for 5, 10, and 30 days (*n* = 4 tanks per treatment). Catalase (CAT) and superoxide dismutase (SOD) activity, glutathione levels, and the alkaline comet assay (% DNA in tail) were analyzed. CAT increased significantly at all concentrations and timepoints, with 80.6% maximum induction at 2.0 mg/L on Day 30 and a LOEC of 0.02 mg/L. SOD showed no significant response at any timepoint. The levels of glutathione increased at Day 5 for the two highest concentrations, but decreased at Day 10 for 3.0 mg/L. Genotoxic effects were significant at Days 5 and 10, with a threshold between 0.02 and 2.0 mg/L. A significant positive correlation between group mean CAT activity and % DNA in the tail contrasted with a null SOD–DNA association, identifying hydrogen peroxide, not superoxide, as the proximate genotoxic driver in adult cnidarian tissue. These results indicate that oxybenzone induces oxidative stress responses in *A. pallida*.

## 1. Introduction

Coral reefs are among the most productive and economically significant ecosystems on Earth, sustaining fisheries, providing coastal protection, and harboring an estimated one-quarter of all marine species, despite occupying less than 1% of the ocean [1,2]. However, climate change-related thermal stress can pose a threat to reef-building corals [3]. For example, from 2015 to 2016, the third global bleaching event produced mortality of unprecedented spatial extent across the Indo-Pacific, Caribbean, and Red Sea [4]. In addition to thermal stress, long-term declines in coral reefs have been linked to overfishing, eutrophication, and chemical pollution, which have further eroded the capacity of reefs to recover between bleaching events [5]. Between January 2023 and March 2025, bleaching-level heat stress affected 84% of the world’s reef area across 82 countries, marking the fourth most spatially extensive global event in recorded history [6,7]. Although effects of thermal stress have been well-studied in corals, other stressors like xenobiotics have received less attention. One class of emerging contaminants that pose a threat to coral reefs are organic UV filters (“sunscreens”).

Oxybenzone (benzophenone-3; BP-3) is the most thoroughly studied organic UV filter in the context of coral ecotoxicology [8]. It enters reef waters principally from recreational swimmers and coastal wastewater. Seawater concentrations range from the low nanograms per liter range at remote sites to 1.4 mg/L at high-traffic beach locations [9]. Danovaro et al. [10] were among the first to report in situ coral bleaching attributable to sunscreen compounds, demonstrating that UV filter concentrations at levels measured in recreational reef zones were sufficient to activate latent viral infections in zooxanthellae. Subsequent analytical surveys detected BP-3 in coral tissues at concentrations of 2.8 to 241 ng/g dry weight [11,12]. Beyond swimmer shedding, beach shower discharge at intensively used marine protected areas constitutes an additional point source for BP-3 contamination. Downs et al. [13] found that oxybenzone concentrations in the sand and water adjacent to beach shower outlets exceeded risk quotients at multiple Hawaiian sites. Hydrodynamic modeling of Hanauma Bay in Hawaii confirmed that this contamination can persist in semi-enclosed reef water for 14 to 50 h following a single shower-associated input [14]. As a result, regulatory pressure has intensified alongside scientific evidence, eventually leading in September 2025 to the European Chemicals Agency classifying BP-3 as an endocrine disruptor for both human health and the environment, concluding a ten-year evaluation [15].

One mechanism by which BP-3 damages cnidarians was investigated by Vuckovic et al. [16], who demonstrated that sea anemones and corals convert oxybenzone to phototoxic glucoside conjugates through constitutively expressed glycosyltransferases. Under UV irradiation, these conjugates function as strong photo-oxidants, generating hydrogen peroxide and singlet oxygen within cnidarian cells. Animals lacking their algal symbionts (aposymbiotic) accumulated higher intracellular conjugate concentrations and died faster than symbiotic counterparts, an observation with direct relevance to bleached corals in the field [16]. Earlier work with the structurally related compound, benzophenone-2, documented comparable bleaching, planula deformation, and genotoxicity in *Stylophora pistillata* [17]. Comparisons across four benzophenone UV filters also found that adult corals are more sensitive to BP-3 than larvae, with LOEC values for BP-3 in the micrograms-per-liter range [18].

In addition to mortality, BP-3 may also induce oxidative stress in aquatic organisms [19,20]. Oxidative stress may have sublethal consequences that jeopardize coral health, and heighten their vulnerability to stressors [21], which has been implicated at a mechanism in coral bleaching [22]. Oxidative stress induced by chemical exposure has been well documented in many marine species [21,23,24,25,26], including corals and other cnidarians [27,28,29,30,31], specifically *Aiptasia pallida* [27]. In fact, sublethal oxidative stress responses can gradually reduce resilience to stressors and may occur before obvious bleaching or tissue loss [32]. Therefore, depending only on bleaching or survival endpoints may underestimate the ecological impact of oxybenzone exposure, particularly in situations involving recurrent or chronic low-level exposure.

There are cellular defenses against oxidative stress, which include catalase and superoxide dismutase. These enzymes are the main enzymes of antioxidant defense against hydrogen peroxide and superoxide, respectively. Because each enzyme responds selectively to a different reactive oxygen species, measuring them simultaneously provides a diagnostic fingerprint of the oxidative pathway responsible for toxicity [24]. These enzymes have been found to be involved in oxidative stress responses in corals [27,33].

Another important intracellular antioxidant is glutathione (GSH). In addition to acting as a substrate for glutathione-dependent enzymes involved in peroxide reduction and xenobiotic detoxification, GSH directly contributes to the neutralization of reactive oxygen species [34]. Variations in GSH levels are frequently employed as sensitive biomarkers of oxidative stress in a variety of species and reflect shifts in cellular redox balance [34]. While GSH depletion may occur under severe or prolonged stress as consumption surpasses regeneration, GSH pools may rise under moderate stress due to compensatory synthesis [34]. Thus, GSH is a mechanistically informative signal of cellular stress that connects molecular reactions and organism-level effects. GSH has also been shown to increase in response to oxidative stress in corals [33] and *A. pallida* [34], and thus is a physiologically significant indicator of oxidative stress in this group of animals.

Besides antioxidant defenses, oxidative stress markers can include molecular damage, such as oxidative DNA damage [35]. Oxidative DNA damage has been observed in many marine species [36], including corals [29,30,31,37]. One method of assessing oxidative DNA strand breakage is the alkaline comet assay, which detects single-strand DNA breaks and alkali-labile sites in individual cells, and has been used extensively in aquatic ecotoxicology as a sensitive indicator of genotoxic exposure [38,39,40]. This assay has been successfully used in assessing genotoxicity in cnidarians [37]. In fact, benzophenone has been shown to be genotoxic in corals [9]. Therefore, combining antioxidant biomarkers with genotoxicity and whole-organism endpoints in a chronic exposure study addresses an important data gap identified in recent critical reviews of UV filter hazard assessment [8].

One model organism used to investigate this is the sea anemone, *Aiptasia pallida* (Verrill, 1864). This organism has become well-established as a cnidarian model organism for coral reef biology. It possesses a fully annotated genome [41], documented antioxidant physiology [42], and has been shown to be responsive to toxicity from metal–organic contaminants [43,44,45,46]. Aposymbiotic (symbiont-free) cnidarians accumulate higher concentrations of oxybenzone-derived glucoside conjugates [16], placing this strain in a position analogous to thermally bleached field corals.

However, there is a paucity of information on the effects of oxybenzone on oxidative stress in corals and *A. pallida*. Such information would provide valuable insight into the mechanisms of chemically induced toxicity and bleaching in corals exposed to oxybenzone. Therefore, the objective is to examine oxidative stress markers in *A. pallida* exposed to oxybenzone. The present study quantified CAT and SOD activity, comet assay genotoxicity, and GSH concentrations in adult *A. pallida* across four oxybenzone exposure concentrations (0.02, 0.2, 2.0, and 3.0μg/L) and three exposure durations (Days 5, 10, and 30). Dual correlation analyses between enzyme activity and DNA damage were used to identify which reactive oxygen species mediate oxybenzone’s genotoxicity in adult cnidarian tissue. The hypothesis was that BP-3 induces oxidative stress in *A. pallida*, as reflected by levels of catalase and superoxide dismutase activity, DNA strand breakage, and GSH.

## 2. Materials and Methods

### 2.1. Chemicals and Reagents

Oxybenzone (benzophenone-3; BP-3; ≥98% purity; CAS No.131-57-7) was purchased from Sigma-Aldrich (St. Louis, MO, USA). Analytical purity was confirmed by the supplier certificate of analysis. A primary stock solution at 5 g/L was prepared in HPLC-grade acetone (Sigma-Aldrich, St. Louis, MO, USA; ≥99.5% purity) and stored at 4 °C in amber glass vials for up to 14 days. Working stocks were prepared fresh on each exposure day by serial dilution of the primary stock in Milli-Q ultrapure water (18.2 MΩ·cm; Millipore Milli-Q, Darmstadt, Germany), and then added to artificial seawater at the appropriate volume to achieve nominal concentrations of 0.02, 0.2, 2.0, and 3.0 mg/L. The acetone carrier concentration was matched across all treatment groups and maintained at or below 0.01% *v*/*v* in every tank, a concentration that produces no measurable effects in aquatic invertebrate assays [47].

Artificial seawater was prepared by dissolving Instant Ocean^®^ Sea Salt (Spectrum Brands, Richmond, VA, USA) in reverse osmosis water to a target salinity of 33 to 35 ppt and aerating for at least 24 h before use. Enzyme assay kits comprised the Novus Biologicals Catalase Activity Assay Kit (catalogue number NBP3-24477; ammonium molybdate colorimetric method; Novus Biologicals, Centennial, CO, USA) and the Sigma-Aldrich SOD Activity Assay Kit (catalogue number CS0009; WST-1 dye inhibition method; Sigma-Aldrich). Protein concentration was determined using the Thermo Scientific Pierce Bradford Protein Assay Kit (catalogue number 23200; bovine serum albumin standards; Thermo Scientific, Waltham, MA, USA). All other reagents were of analytical grade or higher and purchased from Sigma-Aldrich, St. Louis, MO, USA unless stated otherwise.

### 2.2. Test Organism and Acclimation

*A. pallida* was obtained from Addictive Reef Keeping (Marathon, FL, USA) and transported to the laboratory in temperature-controlled coolers. Upon arrival, specimens were transferred individually to 20 L glass aquaria filled with aerated artificial seawater and allowed to settle for 24 h before feeding commenced. The acclimation period lasted seven days at 25 ± 1 °C under a 12 h light and 12 h dark photoperiod delivered by cool-white fluorescent ceiling lighting and Coralife Seascape^®^ LED aquarium lights (Central Aquatics, Franklin, WI, USA) suspended above the tanks. This arrangement produced an irradiance of approximately 50 μmol photons m^−2^ s^−1^, measured with a calibrated LI-COR LI-190R quantum sensor (Apogee Instruments, Inc., Logan, UT, USA) at the water surface. Animals were fed freshly hatched *Artemia* sp. nauplii *ad libitum* three times per week throughout both acclimation and the exposure period. Uneaten food and waste were removed by gentle pipette aspiration within six hours of each feeding event. Only individuals that extended their tentacles fully and actively captured prey during the final 48 h of acclimation were selected for the experiment. Animals were also checked for visible signs of stress or retraction on the morning of tank assignment, and any individual that remained contracted was excluded. Body size was not standardized, but wet masses at Day 0 (recorded at the time of tank assignment) showed no significant differences among treatment groups (one-way ANOVA: F(5,642) = 0.83, *p* = 0.53), confirming balanced initial conditions.

### 2.3. Experimental Design and Exposure

Twenty-four 8 L glass aquaria were used as experimental units. The six treatment levels (negative control, solvent control, and oxybenzone concentrations of 0.02, 0.2, 2, and 3 mg/L) were each replicated in four tanks, giving *n* = 4 independent tank replicates per treatment. Tank positions were assigned using a random number sequence generated in R. Twenty-seven *A. pallida* were allocated to each tank using simple random assignment from a pooled acclimated population, with the constraint that no more than one animal came from the same source container per tank. This gave 648 animals in total, sufficient to provide three animals per tank at each of the three sacrifice dates (Days 5, 10, and 30) without resampling individuals.

Exposure solutions were prepared each morning at 07:00 by dissolving the BP-3 working stock in freshly prepared artificial seawater. Half of the water in each tank was emptied and refilled with fresh artificial seawater once every 24 h throughout the 30-day study, following a static-renewal protocol. Additional oxybenzone stock was added after water changes to maintain the concentration. This renewal interval was selected to approximate the pseudo-persistent nature of oxybenzone contamination in marine recreational environments while ensuring that ammonia and metabolite accumulation remained within acceptable limits. Tank position was rotated weekly in a Latin-square pattern to eliminate any location effects. The experiment was conducted without UV supplementation above ambient laboratory fluorescent light, a conservative design chosen to assess the direct effects of oxybenzone exposure independently of its UV-activated phototoxicity pathway, as previously established by Vuckovic et al. [16].

### 2.4. Water Quality Monitoring

Temperature and dissolved oxygen of the water were measured daily using a calibrated GIDIGI dissolved oxygen meter (accuracy ±0.1 mg/L for DO; ±0.1 °C for temperature; JiNan Huiquan Electronic Co., Ltd., Jinan, China). Salinity was measured by digital refractometry using a hand-held meter (Orapxi International, Tianjin, China) before every water change. The pH was monitored with an Extech EC500 multiparameter meter (Extech Instruments, Nashua, NH, USA) calibrated against pH 7.0 and 10.0 buffer solutions at the start of each measurement session. Conductivity, which was used as an additional stability check for salinity consistency, was recorded alongside pH with the Extech meter. All physical parameters were recorded between 08:00 and 09:00 to minimize diurnal variation in dissolved oxygen readings. Total ammonia was quantified using a salicylate-based colorimetric assay based on the API ammonia test kit (API Fishcare, Chalfont, PA, USA), modified for use in a 96-well microplate format. After mixing aquarium water with the reagents provided in the kit, ammonium was quantified by absorbance at 680 nm using a BioTek plate reader (BioTek Instruments, Inc., Winooski, VT, USA). A standard curve was constructed from twelve ammonium chloride standards ranging from 0.0039 to 8 mg/L in deionized water on Days 1, 5, 10, 20, and 30. If any parameter fell outside the predetermined acceptable range for *A. pallida* culture on two consecutive measurement days, the protocol required immediate investigation and corrective action before continuing. In practice, no such event occurred during this study.

### 2.5. Oxybenzone Concentration Verification by LC-MS/MS

Water samples of 1 mL were collected from each of the 24 tanks on Days 1, 5, 10, 20, and 30 immediately before the daily tank renewal. Samples were collected into pre-cleaned 2 mL amber HPLC vials, capped immediately, and stored at 4 °C. Analysis was completed within 48 h of collection at the Shimadzu Innovation Lab, Department of Chemistry, SIUE. Samples were diluted ten-fold with 50% *v*/*v* acetone in Milli-Q water and passed through a 0.45 μm PVDF syringe filter before injection.

Nine calibration standards were prepared at concentrations from 1 ppb to 1 ppm BP-3 by diluting a 1 ppm stock standard with 50% *v*/*v* acetone in Milli-Q water using a Shimadzu AOC-6000 Plus Multifunctional Autosampler (Shimadzu Scientific Instruments, Baltimore, MD, USA). All BP-3 analyses were conducted on a Shimadzu LCMS-8060NX (Shimadzu Scientific Instruments, Columbia, MD, USA) triple quadrupole mass spectrometer in multiple reaction monitoring (MRM) mode. Chromatographic separation used a HALO Reverse Phase-Amide column (2.7 μm particle size, 4.6 × 100 mm, Advanced Materials Technology, Wilmington, DE, USA) with the column oven temperature held at 40 °C. A binary gradient was used that consisted of 5% LCMS-grade acetonitrile and 0.1% formic acid (mobile phase A) and 0.1% formic acid in 95% LCMS-grade acetonitrile (mobile phase B). At a constant flow rate of 1 mL/min, the elution gradient was as follows: 0 to 10 min at 75% B and a 10 to 12 min ramp from 75% to 100% B.

For identification and quantitation, the BP-3 MRMs were monitored in positive mode electrospray ionization (ESI) with the corresponding pre-rod bias and optimized collision energies as listed in Table 1. Additional instrumental parameters included a nebulizing gas flow of 3 L/min, a heating gas flow of 15 L/min, an interface temperature of 400 °C, a desolvation temperature of 650 °C, a desolvation line temperature of 250 °C, a heat block temperature of 400 °C, and a drying gas flow of 3 L/min. Data were collected at a sampling rate of 5 Hz. BP-3 concentrations were calculated based on an external calibration curve generated through LabSolutions Insight software (Version 5.128, Shimadzu Scientific Instruments, Columbia, MD, USA).

Nominal concentrations are used throughout the text when referencing treatment groups.

### 2.6. Biological Sampling and Wet Tissue Mass

Sampling was conducted between 10:00 and 12:00 CST on each sampling day to minimize circadian effects on enzyme activity. Three *A. pallida* were removed from each of the 24 tanks per sampling event (72 animals total per timepoint). Each animal was transferred individually to a clean 100 mL glass beaker of fresh artificial seawater, rinsed twice by transfer to further clean beakers, and then blotted gently by placing the animal on pre-weighed Kimwipe tissue for exactly 30 s. Wet mass was recorded immediately on a Mettler-Toledo MS205DU analytical balance (±0.0001 g, Mettler-Toledo, Columbus, OH, USA). Animals destined for enzyme and GSH assays were snap-frozen in liquid nitrogen within 30 s of weighing and transferred to pre-labeled 2 mL cryogenic tubes stored at −80 °C. Each frozen sample was processed for both CAT and SOD from the same tissue aliquot, with sub-samples taken after homogenization. Animals designated for the comet assay were placed on ice in Ca^2+^/Mg^2+^-free phosphate-buffered saline containing 20 mM EDTA (pH 7.4) and processed within two hours of collection. The tank mean of three animals served as the biological replicate for all downstream analyses, providing *n* = 4 independent tank means per treatment per timepoint and preventing pseudoreplication [48].

### 2.7. Tissue Homogenization and Protein Quantification

For catalase assays, each frozen tissue sample was weighed on the analytical balance and homogenized at approximately 10% *w*/*v* in ice-cold 0.01 M phosphate-buffered saline (pH 7.4) containing 1 mM EDTA. Homogenization was performed with an IKA T10 Ultra-Turrax (IKA Works, Inc., Wilmington, NC, USA) disperser at 20,000 rpm in three 10 s bursts, with 30 s cooling intervals on ice between bursts. The homogenate was centrifuged at 10,000× *g* for 10 min at 4 °C. The resulting supernatant was diluted 1:5 in assay buffer before enzyme activity measurement.

For SOD assays, a separate tissue aliquot from the same animal was homogenized in ice-cold 0.1 M Tris-HCl buffer (pH 7.4) containing 0.5% Triton X-100, 5 mM β-mercaptoethanol, and a commercial protease inhibitor cocktail at 1× working concentration. The buffer-to-tissue ratio was approximately 10 μL per milligram of wet tissue. The homogenate was centrifuged at 14,000× *g* for 5 min at 4 °C. The supernatant was diluted 1:10 in the assay working solution before analysis.

For GSH determination, animals were homogenized in a 5% sulfosalicylic acid (SSA) solution in Milli Q water. To reduce oxidative degradation, tissue homogenization was carried out on ice at a tissue-to-homogenization solution ratio of 1:10 (*w*/*v*). Homogenates were centrifuged at 14,000× *g* for 10 min at 4 °C. GSH analysis was performed using the resultant supernatants. When feasible, samples were analyzed immediately; if not, aliquots were kept at −80 °C until analysis.

### 2.8. Catalase Activity Assay

Catalase activity was measured in duplicate using the Novus Biologicals NBP3-24477 kit, which quantifies the residual hydrogen peroxide remaining after a fixed-duration enzymatic reaction. Each assay well received 20 μL of diluted tissue supernatant and 30 μL of 100 mM H_2_O_2_ in 50 mM potassium phosphate buffer (pH 7.0), giving an initial substrate concentration of 43 mM H_2_O_2_ per well after mixing. The reaction proceeded for exactly 1 min at 37 °C. Addition of 50 μL of ammonium molybdate reagent stopped the reaction and formed a yellow phosphomolybdate complex proportional to residual H_2_O_2_. Absorbance was read at 405 nm on a BioTek Synergy H1 multimode plate reader (BioTek Instruments, Winooski, VT, USA). A fresh H_2_O_2_ standard curve (0 to 100 μmol/mL; eight points) was prepared for each plate. No more than eight tissue samples were included on any single plate to ensure that all unknowns fell within the linear range of the standard curve. One unit of catalase is defined as the amount of enzyme that decomposes 1 μmol of H_2_O_2_ per minute at 37 °C, and activity is reported as U/mg protein.

### 2.9. Superoxide Dismutase Activity Assay

SOD activity was quantified in duplicate by the xanthine/xanthine oxidase WST-1 inhibition method (CS0009 kit; Sigma-Aldrich (St. Louis, MO, USA)). In this system, xanthine oxidase catalyzes superoxide production from xanthine, superoxide subsequently reduces the tetrazolium salt WST-1 to a soluble formazan dye, and the absorbance is measured at 450 nm. Superoxide dismutase in the tissue sample competes with WST-1 for superoxide, causing a decrease in dye reduction that is proportional to SOD activity. All working solutions were prepared fresh on the day of assay, kept on ice, and protected from light until use. The assay format was 200 μL per well in the assay plate: 20 μL of diluted sample, 160 μL of WST-1 working solution, and 20 μL of xanthine oxidase solution. Plates were incubated at 20 to 25 °C for 30 min in the dark before reading at 450 nm. SOD standards were prepared from the kit standard (300 U/mL stock) in 0.1 M Tris-HCl buffer across the range 0 to 6 U/mL at seven concentrations. The rate of WST-1 reduction was linearized as the linear substrate rate (LSR = (A_0_ − A_s_)/A_s_) before interpolation against the standard curve. Activity is expressed as U/mg protein, where one unit is the amount of SOD producing 50% inhibition of WST-1 reduction under the assay conditions.

### 2.10. Genotoxicity Assessment: Alkaline Comet Assay

DNA strand breakage was assessed using the alkaline single-cell gel electrophoresis assay at pH > 13, following Singh et al. [39] and updated guidelines of Tice et al. [49] and Collins et al. [50], with modifications developed in our laboratory for cnidarian tissue. All steps were performed either under yellow-filtered safelight or in complete darkness to prevent UV-induced DNA damage during processing.

Each animal was placed in 0.5 mL of ice-cold Ca^2+^/Mg^2+^-free phosphate-buffered saline containing 20 mM EDTA and minced finely with iridectomy scissors for 60 s. The resulting cell suspension was passed through a 70 μm cell strainer to remove tissue fragments, and cell density was estimated by a hemocytometer (Neubauer improved chamber) and adjusted to 1 × 10^5^ viable cells per milliliter. Cell viability was confirmed by trypan blue exclusion (≥90% viability required for inclusion). Fifty microliters of cell suspension were mixed with 450 μL of 0.5% low-melting-point agarose (LMA; BioReagent grade; Sigma-Aldrich, St. Louis, MO, USA) held at 37 °C, giving a final agarose concentration of 0.45%. This mixture was layered onto two CometSlide™ wells per sample (Trevigen, Gaithersburg, MD, USA), pre-coated with 0.5% normal-melting-point agarose (NMA). Slides were placed on a flat metal baking tray at 4 °C for 10 min to allow for gel solidification before immersion in freshly prepared lysis solution (2.5 M NaCl, 100 mM EDTA, 10 mM Tris, 1% Triton X-100, 10% DMSO; pH 10, adjusted with 10 M NaOH; 4 °C; 60 min).

After lysis, slides were transferred to an alkaline electrophoresis tank pre-filled with freshly made alkaline unwinding solution (300 mM NaOH, 1 mM EDTA; pH > 13) and allowed to unwind for 20 min at 4 °C. Electrophoresis was then conducted in the same buffer at 21 V (1 V/cm) and approximately 300 mA for 30 min. Slides were removed and washed three times for 5 min each in 0.4 M Tris-HCl (pH 7.5) to neutralize residual alkali. After a brief rinse in absolute ethanol, slides were air-dried completely at room temperature and stored in darkness until staining. Immediately before scoring, slides were flooded with 100 μL of SYBR Green I working solution (1:10,000 dilution in 10 mM Tris-EDTA buffer; pH 7.5) and incubated for 30 min at room temperature in a humidity chamber protected from light. Excess stain was drained and slides were covered with a glass coverslip.

Comet images were acquired on a Leica DM5500B epifluorescence microscope (Leica Microsystems, Wetzlar, Germany) equipped with a FITC filter set (excitation 494 nm, emission 521 nm) and a Leica DFC7000T digital camera (Leica Microsystems, Wetzlar, Germany), using Leica Application Suite v3.2.0. Images were captured at 20× objective magnification. A minimum of 50 cells per replicate (two slides combined) were scored using OpenComet v1.3.1 [51] as a plug-in for ImageJ (Fiji distribution, version 2.14.0). The plug-in was configured to exclude ghost cells (tail DNA fraction ≥ 90%), cells intersecting the gel boundary, and overlapping nuclei. The operator scoring each slide was masked to treatment identity. Percentage DNA in the tail was selected as the primary genotoxicity metric because it is robust to variation in staining intensity and provides a continuous, normally distributed response variable in well-designed comet experiments [52]. All slides from each timepoint were processed in a single batch to ensure uniform lysis, electrophoresis, and staining conditions across treatment groups.

### 2.11. Glutathione Assay

GSH concentrations were determined according to Theodorakis et al. [53]. Briefly, the supernatant was allowed to react with 10 μL of naphthalene-2,3-dicarboxaldehyde (10 mM in water). This process creates a fluorescent product by selectively reacting with reduced GSH. A microplate reader equipped with a 472/528 nm excitation/emission filter set was used to measure fluorescence. Reduced GSH standards at concentrations of 0.01, 0.025, 0.05, 0.1, 0.25, 0.5, 0.75, and 1 mM were used to create a standard curve. The standard curve was used to compute the GSH concentrations in the samples, which were then reported as nmol GSH per mg protein.

### 2.12. Protein Determination

Protein concentrations in tissue homogenates were determined with the Bradford assay [54], using a commercially available kit (Peirce Chemical Co., Waltham, MA, USA).

### 2.13. Statistical Analysis

Statistical analyses were conducted in R v4.4.2 [55] using the packages nlme (version 3.1-164; linear mixed-effect models), FSA (Dunn’s post hoc test), DescTools (Dunnett’s and Games–Howell tests), onewaytests (Welch’s ANOVA), and coin (permutation tests for confirmatory analysis). The biological replicate throughout was the tank mean calculated from three animals per tank, which gives *n* = 4 independent replicates per treatment group per timepoint and avoids pseudoreplication [49]. Normality of model residuals was checked with the Shapiro–Wilk test and homogeneity of group variances was checked with Levene’s test before each parametric analysis. The significance threshold was α = 0.05 for all tests.

Catalase activity and GSH levels were analyzed using a three-way linear mixed-effect model with Treatment (six levels), Day (three levels; treated as ordered factor), and the Treatment × Day interaction as fixed effects, and Tank nested within Treatment as a random intercept. Denominator degrees of freedom were estimated by the Satterthwaite approximation. Pairwise comparisons between each oxybenzone concentration and the solvent control within each timepoint used Dunnett’s test with family-wise error rate control. SOD activity failed the normality assumption on raw values at all timepoints (Shapiro–Wilk: all *p* < 0.001) and was therefore analyzed by Kruskal–Wallis H test at each timepoint separately, followed by Dunn’s pairwise test with Bonferroni correction. A secondary LME model on log-transformed SOD data was fitted as a sensitivity check and produced identical conclusions.

Comet assay data at Days 5 and 30 met both normality and variance homogeneity assumptions and were analyzed by one-way ANOVA with Dunnett’s post hoc. At Day 10, Levene’s test indicated significantly unequal variances (F(5,18) = 7.308, *p* = 0.001); so, Welch’s heteroscedastic ANOVA was used instead, with Games–Howell pairwise comparisons. Kruskal–Wallis tests with epsilon-squared (ε^2^) effect size estimates served as nonparametric confirmation for all comet results. Wet tissue mass was analyzed by one-way ANOVA (all timepoints combined) with Tukey’s HSD for pairwise comparisons. Pearson’s and Spearman’s correlation coefficients were calculated between the group mean CAT or SOD activity and the group mean percentage DNA in the tail, using all 18 group means formed by crossing the six treatments with the three timepoints. Normality of each correlation variable was checked with the Shapiro–Wilk test before applying the Pearson test. All reported p-values are two-tailed.

All statistical analyses were performed in R version 4.4.2 [55], with a significance threshold of α = 0.05 throughout. The biological replicate in all analyses was the tank mean derived from three animals per tank, giving *n* = 4 independent replicates per treatment group per timepoint and preventing pseudoreplication [48]. Prior to each parametric test, residual normality was assessed with the Shapiro–Wilk test and homogeneity of variance was assessed with Levene’s test.

## 3. Results

### 3.1. Water Quality and Exposure Concentration Verification

All physicochemical water quality parameters remained within acceptable ranges throughout the 30-day study and did not differ significantly among the six treatment groups at any monitoring point (Table 2). Temperature, pH, dissolved oxygen, salinity, and ammonia concentrations were stable across the exposure period, confirming that the experimental environment was not a confounding source of biological variation.

Oxybenzone concentrations measured by LC-MS/MS in each tank across all five monitoring days are given in Table 3. Concentrations in the control and solvent control tanks were at or below the limit of detection. In the 0.02 mg/L nominal group, all measured values fell below the limit of quantification. The 0.2 mg/L nominal group yielded measured concentrations of 84 to 141 μg/L (42 to 71% of nominal), indicating some partitioning to the tank surface over the 24 h exposure interval. Measured concentrations in the 2.0 and 3.0 μg/L nominal groups exceeded nominal values substantially, reaching 5.02 to 6.57 μg/L and 6.10 to 7.76 μg/L, respectively. This pattern is consistent with desorption of BP-3 adsorbed to the glass tank walls during the preceding 24 h exposure cycle, which has been documented for hydrophobic organic UV filters in glass containment systems. All biological responses are described relative to nominal concentration groups throughout this paper. Measured concentrations are provided for transparency and will inform the interpretation of absolute effect levels.

### 3.2. Wet Tissue Mass

Wet tissue mass is reported in Table 4.

### 3.3. Catalase Activity

**Figure 1:**
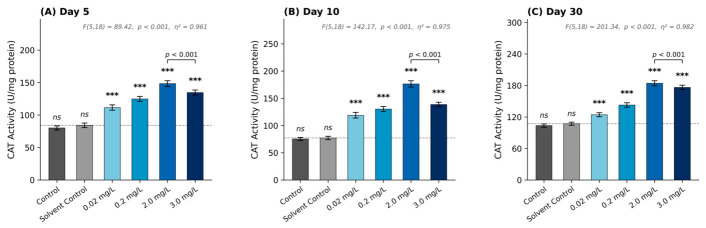
Mean (±SE) catalase (CAT) activity (U/mg protein) in Aiptasia pallida at (**A**) Day 5, (**B**) Day 10, and (**C**) Day 30 (*n* = 4 tank means per treatment). Dashed line indicates the solvent control mean. *** denotes *p* < 0.001 versus the solvent control (Dunnett’s post hoc test); ns = not significant. The 3 mg/L group is consistently below the 2.0 mg/L group at all three timepoints, producing a nonmonotonic dose response. Nominal oxybenzone concentrations in μg/L.

Treatment group, exposure duration, and their interaction were all highly significant in the linear mixed-effect model (Treatment F(5,18.0) = 1209.715; Day F(2,180.0) = 1909.297; Treatment × Day F(10,180.0) = 126.643; all *p* < 0.001; Figure 1). Catalase activity in the negative control and the solvent control did not differ from each other at any of the three timepoints (Dunnett’s: all *p* > 0.05), which confirmed that the acetone carrier did not affect enzyme activity. At every timepoint, CAT activity was significantly elevated above the solvent control in all four oxybenzone groups (Dunnett’s: all *p* < 0.001).

The response progressed steadily from Day 5 through Day 30 in every treatment group, indicating that H_2_O_2_ accumulation was sustained rather than transient (Figure 1). The lowest concentration tested (0.02 mg/L nominal) produced a 26.2% increase above the solvent control on Day 5, rising to 23.0% on Day 30, which established 0.02 mg/L as the lowest observed effect concentration for this endpoint. Maximum induction was recorded in the 2.0 mg/L group on Day 30 (187.10 ± 1.69 U/mg protein versus 103.59 ± 0.81 U/mg protein in the solvent control; +80.6%). A nonmonotonic dose response was present at all three timepoints: the 3.0 mg/L group consistently showed lower CAT activity than the 2.0 mg/L group (Dunnett’s comparison between these two groups at Day 30: *p* < 0.001).

### 3.4. Superoxide Dismutase Activity

**Figure 2:**
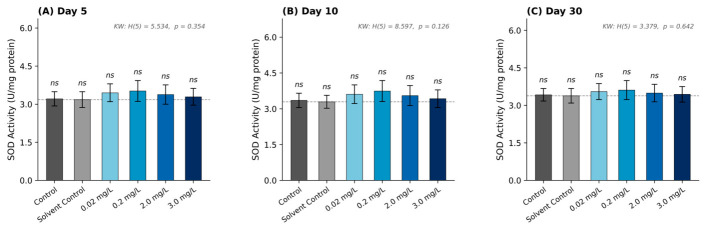
Mean (±SE) superoxide dismutase (SOD) activity (U/mg protein) in *Aiptasia pallida* at (**A**) Day 5, (**B**) Day 10, and (**C**) Day 30 (*n* = 4 tank means per treatment). Dashed line indicates the solvent control mean. Kruskal–Wallis H test statistics and *p*-values are shown within each panel. No significant treatment effect was detected at any timepoint (all *p* > 0.05). Nominal oxybenzone concentrations in μg/L.

SOD activity showed no significant treatment effect at any of the three timepoints (Kruskal–Wallis: Day 5 H(5) = 5.534, *p* = 0.354; Day 10 H(5) = 8.597, *p* = 0.126; Day 30 H(5) = 3.379, *p* = 0.642; Figure 2). Dunn’s pairwise test confirmed that no individual oxybenzone concentration differed significantly from the solvent control at any timepoint after Bonferroni correction. The secondary linear mixed-effect analysis of log-transformed SOD data reached the same conclusion (Treatment F(5,198.0) = 1.338, *p* = 0.250; Day F(2,198.0) = 1.175, *p* = 0.311; interaction F(10,198.0) = 1.240, *p* = 0.268).

### 3.5. Genotoxicity: Alkaline Comet Assay

**Figure 3:**
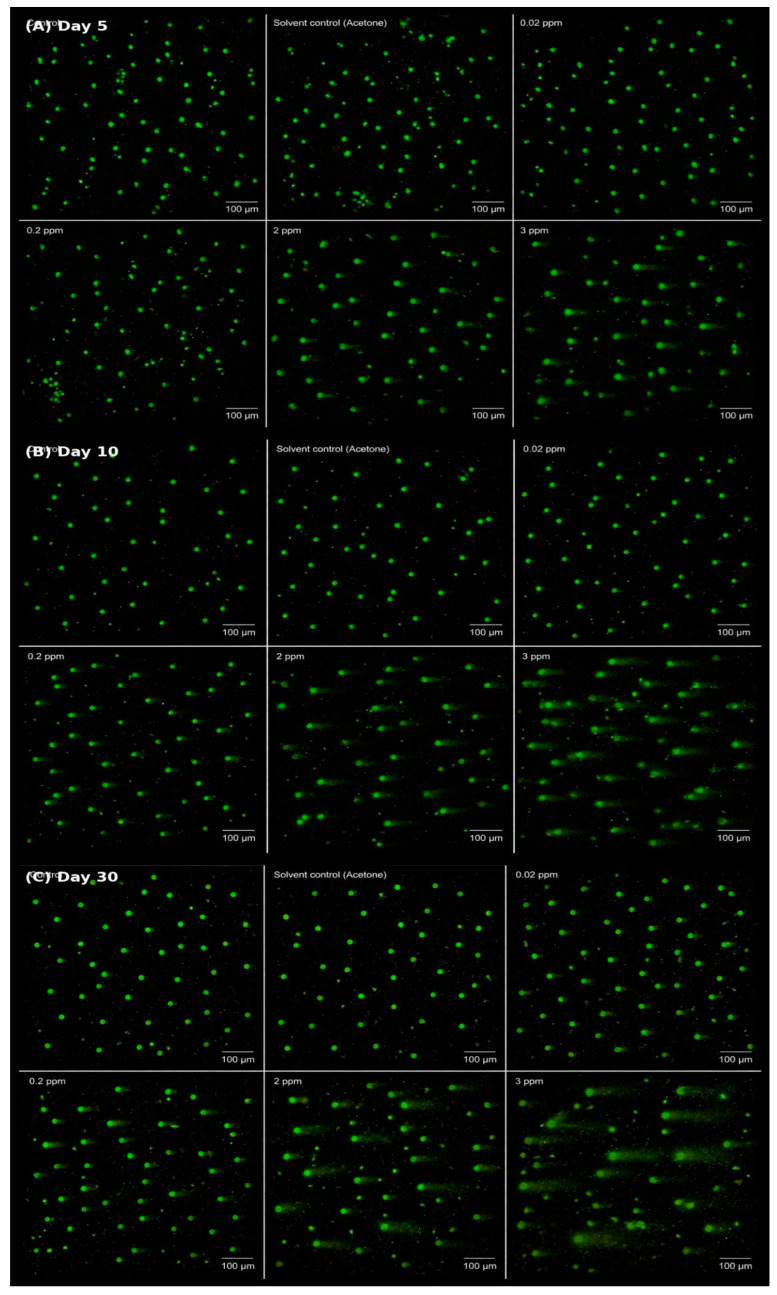
Representative SYBR Green I fluorescence micrographs of comet cells from *Aiptasia pallida* at (**A**) Day 5, (**B**) Day 10, and (**C**) Day 30. Each panel is arranged as a 2 × 3 array: top row shows the control, solvent control, and 0.020 mg/L groups; bottom row shows 0.2, 2.0, and 3.0 mg/L groups. Scale bars = 100 μm. All images are from replicate tank 1 of each treatment group. Nominal oxybenzone concentrations in mg/L.

**Figure 4:**
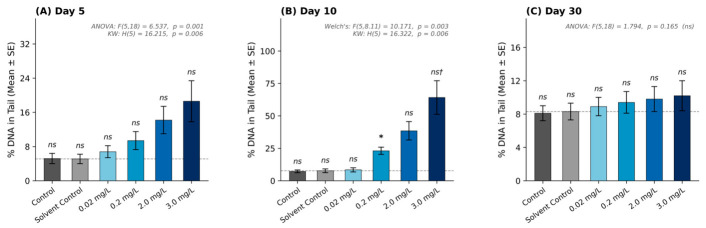
Mean (±SE) percentage DNA in the comet tail in *Aiptasia pallida* at (**A**) Day 5, (**B**) Day 10, and (**C**) Day 30 (*n* = 4 replicate means per treatment; each replicate = mean of at least 50 individually scored cells). Dashed line indicates the solvent control mean. * adjusted *p* < 0.05 versus the solvent control (Games–Howell post hoc, Day 10, 0.2 mg/L group). ns† 0.05 < *p* < 0.10.

Representative fluorescence micrographs of SYBR Green I-stained comet cells from *A. pallida* at each of the three timepoints are shown in Figure 3. Each composite panel displays all six treatment groups. At Day 5, cells from the control groups show compact, circular nuclei with little or no tail formation, while the 2.0 and 3.0 mg/L groups display early tail development visible as a diffuse fluorescent smear extending from the nucleus. The Day 10 panel shows the clearest treatment-related differences: from 0.2 mg/L onwards, elongated tails are readily apparent, and cells in the 3.0 mg/L group include several individuals with extensive DNA migration approaching ghost-cell morphology. By Day 30, tail extension is less pronounced than at Day 10, and cells across all groups appear more similar to one another. Quantitative analysis of these micrographs is presented in Figure 4.

Significant treatment effects on percentage DNA in the tail were detected at Days 5 and 10 but not at Day 30. At Day 5, the one-way ANOVA was significant (F(5,18) = 6.537, *p* = 0.001), corroborated by Kruskal–Wallis H(5) = 16.215, *p* = 0.006 (ε^2^ = 0.705). Dunnett’s post hoc test did not identify any individual concentration as significantly different from the solvent control after family-wise error correction (all adjusted *p* > 0.05), though the large overall effect size indicates that the treatment factor accounted for the majority of variance. At Day 10, Welch’s ANOVA was significant (F(5,8.11) = 10.171, *p* = 0.003; Kruskal–Wallis H(5) = 16.322, *p* = 0.006; ε^2^ = 0.710). Games–Howell post hoc identified the 0.2 mg/L group as significantly elevated above the solvent control (adjusted *p* = 0.035; 23.10 ± 2.77% versus 7.67 ± 1.40%), establishing the genotoxic threshold between 20 and 0.2 mg/L. The 3 mg/L group recorded the highest group mean in the entire study (64.19 ± 12.92%; individual replicate maximum: 87.43%) but narrowly missed the corrected significance threshold (Games–Howell adjusted *p* = 0.097; raw *p* = 0.021), most likely because statistical power at *n* = 4 was insufficient to resolve this comparison against the large within-group variance at this concentration. At Day 30, neither the ANOVA (F(5,18) = 1.794, *p* = 0.165) nor the Kruskal–Wallis test (H(5) = 5.960, *p* = 0.310) reached significance.

### 3.6. Dual Correlation Analyses: CAT and SOD Versus Percentage DNA in the Tail

**Figure 5:**
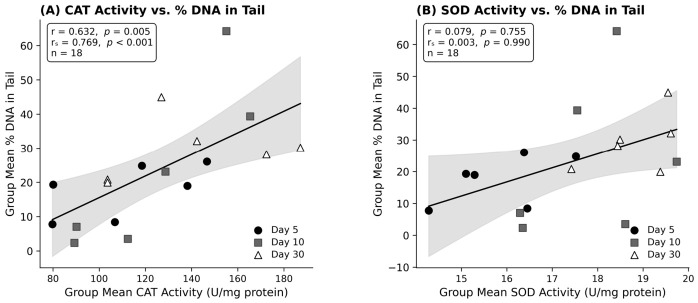
Pearson’s correlation between group mean enzyme activity and group mean percentage DNA in the comet tail across all 18 treatment-by-timepoint group means (*n* = 18). Symbols represent timepoints: circles = Day 5, squares = Day 10, triangles = Day 30. The regression line is shown with a 95% confidence interval band. (**A**) CAT activity: r = 0.632, *p* < 0.05; r_s_ = 0.769, *p* < 0.05 (significant positive association). (**B**) SOD activity: r = 0.079, *p* = 0.755; r_s_ = 0.003, *p* > 0.05 (no association). The shaded area indicates the 95% C.I. The contrasting patterns identify H_2_O_2_ rather than superoxide as the proximate genotoxic driver. The shaded area is the 95% C.I.

The Shapiro–Wilk test confirmed that each of the three correlation variables was normally distributed across the 18 group means (CAT: W = 0.957, *p* = 0.549; SOD: W = 0.955, *p* = 0.501; percentage DNA in tail: W = 0.917, *p* = 0.112). Group mean CAT activity and group mean percentage DNA in the tail were significantly correlated (Pearson’s r = 0.632, r^2^ = 0.400, *p* = 0.005; Spearman’s rs = 0.769, *p* < 0.001; Figure 5A). Roughly 40% of the between-group variation in DNA damage was therefore explained by differences in CAT activity. Group mean SOD activity, in contrast, bore no significant relationship with percentage DNA in the tail (Pearson’s r = 0.079, r^2^ = 0.006, *p* = 0.755; Spearman’s rs = 0.003, *p* = 0.990; Figure 5B). Within-timepoint Pearson’s correlations between SOD and DNA damage were also not significant at any of the three individual days (Day 5: r = −0.241, *p* = 0.630; Day 10: r = 0.261, *p* = 0.604; Day 30: r = −0.107, *p* = 0.836).

### 3.7. Glutathione Analysis

**Figure 6:**
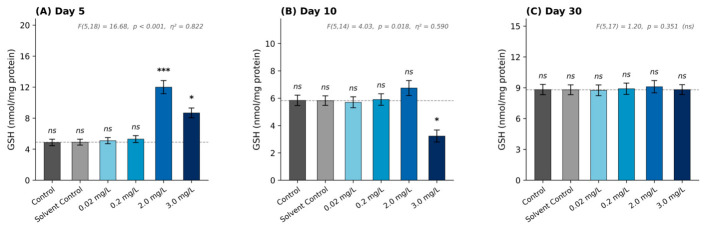
Mean (±SE) glutathione concentrations in *Aiptasia pallida* at (**A**) Day 5, (**B**) Day 10, and (**C**) Day 30 (*n* = 4 replicate means per treatment). Dashed line indicates the solvent control mean. * adjusted *p* < 0.05; *** adjusted *p* < 0.01; ns *p* > 0.05; treatment versus the solvent control (ANOVA with Dunnett’s post hoc test). Nominal oxybenzone concentrations in mg/L.

On Day 5, the one-way ANOVA returned F (5,18) = 16.68 with *p* < 0.001 and an effect size of *η*^2^ = 0.822, meaning that treatment alone explained more than four-fifths of the variance in GSH among groups at this time point. Shapiro–Wilk gave W = 0.947, *p* = 0.236; so, the assumption of normally distributed residuals held. Dunnett’s test then identified which groups were responsible for that effect. Two groups stood out from the untreated control. Anemones exposed to 2.0 mg/L oxybenzone showed a GSH mean of 12.00 nmol/mg protein, fully 7.14 units above control, with t = 6.33 (*p* < 0.001; Figure 6). The 3.0 mg/L group sat at 8.68 nmol/mg protein, 3.82 units above control, t = 3.39 (*p* < 0.05). The two lowest doses, 0.02 and 0.2 mg/L, did not differ from the untreated control by any meaningful margin. The solvent control was likewise indistinguishable from the untreated control, which is a clean validation of the vehicle.

By Day 10, the ANOVA results were still significant but considerably weaker than on Day 5: F (5,14) = 4.03, *p* = 0.018, *η*^2^ = 0.590. Shapiro–Wilk on the residuals returned W = 0.946, *p* = 0.308 (Figure 6). Crucially, only one group now differed from the untreated control by Dunnett’s test, and it differed in the opposite direction. The 3.0 mg/L group had fallen to 3.23 nmol/mg protein, which is 2.61 units below the control mean of 5.84, with t = 3.11 (*p* < 0.05). The 2.0 mg/L group, which greatly exceeded the others on Day 5, was no longer significantly different from control: it had drifted down to 6.74 nmol/mg protein. The 0.02 mg/L group, after correction of the earlier outlier, came in at 5.82, indistinguishable from the control’s 5.84 to two decimal places. Figure 6B shows the results.

At Day 30, the one-way ANOVA results were non-significant: F(5,17) = 1.20, *p* = 0.351, *η*^2^ = 0.261 (Figure 6). Shapiro–Wilk gave W = 0.914, *p* = 0.049, which sits just on the edge of the conventional cut-off and is worth flagging as a minor caveat, but since the test was non-significant the post hoc step would not change the conclusion. No treatment group differed from the untreated control. Most strikingly, the 3.0 mg/L group, which had been so low on Day 10, returned exactly to the control mean of 8.82 nmol/mg protein, the difference being precisely zero.

## 4. Discussion

This study addresses a substantive gap in the oxybenzone literature by providing the first simultaneous quantification of antioxidant enzyme activity, DNA strand breakage, and GSH levels in adult cnidarian tissue across four concentrations and three chronic exposure durations. Catalase activity was elevated at every concentration and every timepoint; SOD was unaffected throughout; genotoxicity was significant at the two shorter timepoints with a clear threshold; and body mass declined with concentration at all timepoints. Taken together, these findings point to a single underlying mechanism: selective intracellular hydrogen peroxide accumulation driving oxidative DNA strand breaks via Fenton chemistry.

### 4.1. Catalase Activity

The pattern of CAT induction in these data is striking for two reasons. First, it is present at the lowest concentration tested. A 0.02 mg/L nominal exposure, below the analytical limit of quantification and therefore representing an unknown but non-zero dose, was sufficient to elevate CAT significantly above the solvent control at all three timepoints. This pushes the LOEC for antioxidant activation well below the concentrations typically used in acute coral toxicity assays and well into the range of seawater concentrations measured near recreational reef sites [9,13]. Second, the activity increased with both concentration and time, reaching its peak at Day 30. Chronic, sustained elevation in CAT in the absence of a corresponding SOD response is consistent with an ongoing, concentration-dependent elevation in intracellular H_2_O_2_. The biochemical mechanism for this was provided by Vuckovic et al. [16], whose identification of oxybenzone-glucoside conjugates as photo-oxidants that produce singlet oxygen under UV irradiation providing a direct upstream source. In the present experiment, the only UV produced would be from fluorescent room lights and the supplemental aquarium lights; so, whether or not this is a sufficient amount of UV to stimulate phototoxic production of H_2_O_2_ by such conjugates remains to be determined. Further investigation is needed to determine if field situations with exposure to solar UV irradiation may produce larger responses. The nonmonotonic dose–response, in which the 3 mg/L group showed lower CAT activity than the 2.0 mg/L group at all timepoints, is a recognized phenomenon in studies of oxidative stress responses, e.g., Refs. [23,25,26,27,28,56]. Catalase is inactivated by its own substrate through compound II accumulation when H_2_O_2_ flux exceeds the regenerative capacity of the enzyme’s heme center [57], but further research is needed to determine if this phenomenon is responsible for the non-monotonic trends seen in the present study.

### 4.2. Superoxide Dismutase Activity

The consistent absence of any SOD response across three timepoints and four concentrations is as informative as the CAT result. Superoxide dismutase catalyzes the conversion of superoxide radical to H_2_O_2_; its induction would be expected if superoxide were accumulating in the cell. The fact that CAT alone is induced is consistent with the hypothesis that the oxidative challenge arises downstream of superoxide, at the H_2_O_2_ step. Work in symbiotic sea anemones has shown that the relative balance of CAT and SOD activities in cnidarian tissue depends on the specific stressor applied, with thermal and chemical stressors producing distinct enzyme response profiles [58,59]. Richier et al. [58] demonstrated that oxidative stress in symbiotic cnidarians can proceed through pathways that activate CAT and glutathione peroxidase without proportionate SOD induction when H_2_O_2_ is the primary ROS. Our data are consistent with the findings of Richier et al. [58] and Nii and Muscatine [60]. They observed analogous patterns in *Aiptasia* under thermal stress, where elevated temperature increased H_2_O_2_-related oxidative damage without obligate superoxide accumulation. There are studies examining oxidative stress responses in *A. pallida* or corals exposed to metals, and these studies also found an increase in catalase but not superoxide dismutase [27,36,61]. Therefore, this might be a generalized pattern in cnidarians exposed to pro-oxidant chemicals.

### 4.3. DNA Damage and Comet Assay

The statistical analysis did not find statistical significance between treatments. However, the fluorescence micrographs in Figure 3 are consistent with the quantitative results at each timepoint. At Day 10, the panel provides a visually direct complement to the statistical findings: comet tails are clearly absent from control cells and progressively more extensive from 0.2 mg/L upwards, with the 3 mg/L group including cells at or near ghost-cell morphology. The partial resolution of DNA damage at Day 30 is worth examining. There are three hypotheses that could explain it. First, cells sustaining extensive strand breaks may be cleared through apoptosis, leaving a surviving population of cells with lower damage [62]. Alternatively, base excision repair capacity could have adapted upward over the 30-day period. A third hypothesis, relevant specifically to the high-dose groups, is that the heavy oxidative burden at these concentrations led to selective mortality of the most vulnerable individuals, altering the composition of the remaining population. The Day 30 results are not negative results; they represent a biologically interesting endpoint that warrants further investigation with molecular markers of DNA repair and apoptosis. In addition, not that the comets in Figure 3 seem to represent two different cell populations: some cells have long tails and some have short/no tails. This may represent the *A. pallida* host cells and their zooxanthyllae symbionts. These two cell populations may have different sensitivities to the toxicant or ROS. This is consistent with the fact that aposymbiotic *A. pallida* (no zooxanthyllae) are more susceptible to oxidative stress than the holosymbiont (with zooxanthyllae), which has been attributed to the zooxanthyllae themselves [58]. This may increase the variation in the comet results, resulting in non-significant results.

### 4.4. The Correlation Between DNA Damage and Enzyme Activity

The paired correlation approach used here offers something that univariate analyses cannot: a direct, quantitative test of which ROS pathway connects antioxidant status to genetic damage. A significant CAT-DNA correlation alongside a null SOD-DNA correlation is precisely the result expected if H_2_O_2_ is the proximate genotoxic species and superoxide is not. Hydrogen peroxide generates hydroxyl radicals through the Fenton reaction (Fe^2+^ + H_2_O_2_ → Fe^3+^ + ·OH + OH^−^). Hydroxyl radicals attack the phosphodiester backbone to produce the strand breaks detected by the alkaline comet assay [58]. To our knowledge, this is the first application of this paired correlation approach in adult cnidarian ecotoxicology. Livingstone [58] reviewed evidence from across aquatic taxa showing that CAT and related peroxidase responses are reliable indicators of H_2_O_2_-mediated genotoxicity in invertebrate tissues, and Valavanidis et al. [61] similarly documented CAT-DNA damage co-variation in marine organisms under oxidant-generating pollutant exposure. Our data add cnidarians to this pattern and provide a cleaner mechanistic case because of the simultaneous null SOD result. However, it should be noted that ROS were not directly measured, and the hypothesis that the DNA damage in this experiment was caused by H_2_O_2_ has not yet been directly tested.

### 4.5. Glutathione Concentrations

The data indicate that short-term exposures lead to an increase in GSH concentrations. This pattern is often seen in many organisms upon exposure to pro-oxidant chemicals [63]. In addition, hyperthermal stress is known to cause oxidative stress in Anthozoans, and Sunagawa et al. [34] found an increase in glutathione levels in *A. pallida* that were exposed to thermal stress for up to 8 days. This is analogous to the findings of the current study, assuming oxybenzone causes increased ROS. Such an increase in GSH may indicate a short-term adaptive response to increased ROS production. The reduction of GSH at the highest concentration after 10 days is consistent with GSH depletion. A similar pattern (initial increase, and reduced levels after chronic exposures) was found by Main et al. [27], where copper exposure in *A. pallida* caused an initial increase in glutathione, followed by decreases after chronic exposure. Sunagawa et al. [34] also found that GSH increased over time in thermally stressed *A. pallida* for up to 3 days of exposure, then leveled off, or even slightly decreased, after 8 days. Thus, an initial increase in GSH followed by a decrease may be a general response to chronic ROS production in this species, no matter the source of ROS induction.

### 4.6. Environmental Relevance and Risk Context

The lowest observed effect concentrations identified in this study (0.020 mg/L for CAT induction; 0.02 to 0.2 μg/L for genotoxicity) fall within the range of seawater concentrations documented at actively used coral reef sites. Downs et al. [9] reported seawater oxybenzone concentrations from 0.075 to 1.4 mg/L at sites in the US Virgin Islands [9]. Concentrations from beach shower discharge zones in marine protected areas exceeded risk quotients of one [13]. He et al. [18] reported adult coral nubbins to be sensitive to benzophenone compounds in the microgram per liter range, consistent with our LOEC estimates. The convergence of our mechanistic toxicology data with the regulatory reclassification of BP-3 as an endocrine disruptor by ECHA in 2025 [15] and the ongoing fourth global bleaching event [6] provides a strong scientific basis for precautionary restrictions on oxybenzone at reef-adjacent sites. These data support incorporating chronic sublethal biomarker endpoints, particularly catalase activity, comet assay genotoxicity, and body mass, into water quality criteria for BP-3 in marine protected area management.

### 4.7. Limitations

Four tank replicates per treatment is a recognized limitation of this study. The statistical power constraint likely explains why the 3.0 mg/L Day 10 comparison narrowly missed the corrected threshold despite a raw *p*-value of 0.021, and why individual pairwise comparisons at Day 5 did not reach significance despite a highly significant omnibus test. Future replication with *n* ≥ 8 tanks per treatment would substantially improve post hoc resolution. The desorption effect documented in Table 3 introduces uncertainty into the actual concentrations experienced by animals in the high-dose groups; a flow-through exposure system would control for this in future work. Finally, the absence of UV irradiation means that the phototoxic amplification pathway identified by Vuckovic et al. was not active in this study; so, all measured effects represent a lower-bound estimate of oxybenzone toxicity under natural field irradiance conditions. Oxybenzone exposure stimulates antioxidant defenses, at least at some timepoints and concentrations. Presumably, this indicates production of reactive oxygen species, but, at present, it is not known if these ROS are produced. In the present study, the increases in DNA strand breakage were not statistically significant for most concentrations, but this may be a limitation of the study design or data analysis, as the distribution of the data suggests there may be a biologically significant effect at higher concentrations on day 5 (Figure 5B). In addition, all endpoints indicated a transient response, with no significant effects on Day 30, which suggests acclimation to oxybenzone toxicity with chronic exposure. However, it is not known if this pattern would still be seen with even longer exposures, e.g., 60 or 90 days. Also, the concentrations used in this study are generally higher than those found in the field. However, field concentrations can reach 0.0279 mg/L [9]; so, the lowest concentration used in this study is within the upper limit of environmental concentrations. Finally, the presence of ROS is inferred by the alterations in enzymes activities or DNA damage, but has not been actually confirmed by measurement of these ROS.

## 5. Future Directions

Besides testing the hypotheses posited in the discussion above, there are also several additional lines of future research that could be pursued, including studies that examine: (1) the expression of these endpoints in actual corals, (2) the expression of antioxidant genes (e.g., catalase, superoxide dismutase, glutathione peroxidase, etc.), (3) indicators of oxidative stress or molecular damage, such as H_2_O_2_ production, lipid peroxidation, protein carbonyls, and oxidative damage to DNA (e.g., 8-hydroxyguanine), (4) the relationship between oxidative stress biomarkers and fitness parameters such as growth, survival, development, and reproduction, (5) the comparison between symbiotic and aposymbiotic (“bleached”) *A. pallida* exposed to oxybenzone, as aposymbiotic animals are more susceptible to oxidative stress [58], (6) interactive effects of oxybenzone and thermal stress in *A. pallida*, as elevated temperatures have been found to induce oxidative stress in corals [62], (7) oxidative stress induced by other sunscreen chemicals, singly or in combination, (8) the above endpoints in planula of *A. pallida* or planula or adults of coral, (9) separating host and zooxanthyllae cells, e.g., [64,65], and performing oxidative stress and comet assays performed separately on the host and zooxanthyllae, and (10) these endpoints in field animals exposed to oxybenzone, determining the effects of UV exposure on oxidative stress.

## 6. Conclusions

Overall, the hypothesis that oxybenzone induces oxidative stress responses was supported, at least for some endpoints, time points, concentrations, and exposure durations. The authors could not find any other studies examining oxybenzone-induced oxidative stress or antioxidant responses in *A. pallida* or corals; so, this is believed to be the first manuscript reporting such effects. This information can contribute to understanding the mechanisms of oxybenzone toxicity, potential approaches for amelioration of this toxicity in the field, and hazard and risk assessments of oxybenzone in corals.

## Figures and Tables

**Table 1:** Oxybenzone Multi-Reaction Monitoring (MRM) Transitions and LC-MS/MS Parameters.

Precursor *m*/*z* ([M^+^H]^+^)	Product *m*/*z*	Dwell Time (msec)	Q1 Pre-Bias (V)	CE (eV)	Q3 Pre-Bias (V)
228.90	151.20	13.0	−15.0	−19.0	−15.0
	105.05	13.0	−15.0	−22.0	−17.0
	95.10	13.0	−21.0	−33.0	−16.0
	77.05	13.0	−22.0	−39.0	−14.0

**Table 2:** Water quality parameters in toxicity tests of oxybenzone in *Aiptasia pallida*.

Parameter	*n*	Mean	SD	Min	Max	Acceptable Range
Temperature (°C)	720	25.10	0.13	24.8	25.5	25 ± 1 °C
pH	720	8.05	0.01	8.01	8.09	7.9–8.2
Salinity (ppt)	720	33.16	0.38	32.0	34.3	30–36
Dissolved oxygen (mg/L)	720	8.33	0.09	8.00	8.64	>6.0
Ammonia (mg/L)	120	0.031	0.012	0.004	0.057	<0.1

Note. No significant differences among treatment groups at any timepoint (one-way ANOVA: all F(5,714) < 0.50, *p* > 0.79). *n* = 720 for temperature, pH, salinity, and dissolved oxygen (24 tanks × 30 days); *n* = 120 for ammonia (24 tanks × 5 monitoring days).

**Table 3:** Measured concentrations of oxybenzone in the aquarium water.

	Measured Concentration (mg/L)					
Treatment (Nominal, mg/L)	Day 1	Day 5	Day 10	Day 20	Day 30	% Nominal ᵃ
Control (0)	<LOD	<LOD	<LOD	<LOD	<LOD	N/A
Solvent Control (0)	0.008 ± 0.0006	0.0026 ± 0.0003	0.0034 ± 0.0012	0.0019 ± 0.0007	0.0031 ± 0.008	N/A
0.02	<LOQ	<LOQ	<LOQ	<LOQ	<LOQ	<LOQ
0.20	0.141 ± 0.026	0.091 ± 0.007	0.091 ± 0.003	0.100 ± 0.006	0.084 ± 0.004	42–71%
2.0	5.109 ± 0.053	5.184 ± 0.031	5.022 ± 0.019	6.569 ± 0.045	6.340 ± 0.038	251–328%
3.0	6.245 ± 0.004	6.266 ± 0.023	6.095 ± 0.027	7.754 ± 0.120	7.655 ± 0.055	203–259%

Note. All measured concentrations in μg/L. LOD (Limit of Detection) = 0.47 μg/L (S/N ratio ≥ 3); LOQ (Limit of Quantitation) = 1.42 μg/L (S/N ratio ≥ 10). ᵃ Percentage of nominal concentration, averaged across the five monitoring days. Exceedance of nominal concentrations in the 2.0 and 3.0 mg/L groups is attributed to desorption of BP-3 from glass tank surfaces during the 24 h exposure interval. [Internal standard and qualifier MRM transition to be confirmed with Tucker Shimadzu Innovation Lab before submission].

**Table 4:** Wet mass (mg) of *Aiptasia pallida* used in toxicity tests of oxybenzone.

Treatment (Nominal)	Day	Mean ± SE (mg)	SD	Min	Max	Median	CV (%)
Control	5	220.7 ± 1.4	4.8	211	227	221	2.19
	10	221.0 ± 1.7	5.9	213	228	222	2.67
	30	222.0 ± 1.4	4.8	211	229	222	2.16
Solvent Control	5	211.2 ± 1.1	4.0	203	216	212	1.88
	10	210.0 ± 1.4	5.0	200	217	211	2.36
	30	216.0 ± 1.6	5.4	206	224	216	2.50
0.02 mg/L	5	197.8 ± 1.4	4.8	193	210	197	2.43
	10	197.3 ± 1.5	5.1	188	204	199	2.60
	30	191.5 ± 1.3	4.6	185	199	190	2.40
0.2 mg/L	5	188.7 ± 1.3	4.6	182	196	188	2.45
	10	184.3 ± 1.5	5.2	173	192	184	2.82
	30	176.6 ± 1.8	6.2	165	190	177	3.49
2 mg/L	5	179.7 ± 1.1	3.9	174	186	181	2.15
	10	174.9 ± 1.7	6.0	160	186	175	3.43
	30	165.2 ± 1.5	5.3	158	173	165	3.22
3 mg/L	5	169.4 ± 1.9	6.4	155	177	171	3.80
	10	166.0 ± 1.4	5.0	158	175	165	3.01
	30	155.0 ± 1.5	5.2	148	166	155	3.35

## Data Availability

Raw data supporting the conclusions of this article will be deposited in a publicly accessible repository (Zenodo at https://zenodo.org or Dryad at https://datadryad.org) before publication, and the dataset DOI will appear in the final published version. Data are available from the corresponding author on reasonable request during peer review.

## Abbreviations

The following abbreviations are used in this manuscript:

ASW	Artificial seawater
BP-3	Benzophenone-3 (oxybenzone)
CAT	Catalase
CV	Coefficient of variation
DMSO	Dimethyl sulfoxide
ECHA	European Chemicals Agency
H_2_O_2_	Hydrogen peroxide
ICRI	International Coral Reef Initiative
KW	Kruskal–Wallis test
LC-MS/MS	Liquid chromatography tandem mass spectrometry
LMA	Low-melting-point agarose
LME	Linear mixed-effect model
LOD	Limit of detection
LOEC	Lowest observed effect concentration
LOQ	Limit of quantification
MRM	Multiple reaction monitoring
NOAA	National Oceanic and Atmospheric Administration
NOEC	No observed effect concentration
OECD	Organisation for Economic Co-operation and Development
ROS	Reactive oxygen species
SOD	Superoxide dismutase
UV	Ultraviolet
